# Permissivity of Primary Human Hepatocytes and Different Hepatoma Cell Lines to Cell Culture Adapted Hepatitis C Virus

**DOI:** 10.1371/journal.pone.0070809

**Published:** 2013-08-05

**Authors:** Francois Helle, Etienne Brochot, Carole Fournier, Véronique Descamps, Laure Izquierdo, Thomas W. Hoffmann, Virginie Morel, Yves-Edouard Herpe, Abderrahmane Bengrine, Sandrine Belouzard, Czeslaw Wychowski, Jean Dubuisson, Catherine Francois, Jean-Marc Regimbeau, Sandrine Castelain, Gilles Duverlie

**Affiliations:** 1 EA4294, Laboratoire de Virologie, Centre Hospitalier Universitaire et Université de Picardie Jules Verne, Amiens, France; 2 Biobanque de Picardie, Centre Hospitalier Universitaire et Université de Picardie Jules Verne, Amiens, France; 3 Institut Pasteur de Lille, Center of Infection & Immunity of Lille (CIIL), F-59019 Lille, France; Inserm U1019, F-59019 Lille, France; CNRS UMR8204, F-59021 Lille, France; Univ Lille Nord de France, F-59000 Lille, France; 4 Département de Chirurgie Digestive, Centre Hospitalier Universitaire et Université de Picardie Jules Verne, Amiens, France; University of Tennessee Health Science Center, United States of America

## Abstract

Significant progress has been made in Hepatitis C virus (HCV) culture since the JFH1 strain cloning. However, developing efficient and physiologically relevant culture systems for all viral genotypes remains an important goal. In this work, we aimed at producing a high titer JFH1 derived virus to test different hepatic cells’ permissivity. To this end, we performed successive infections and obtained a JFH1 derived virus reaching high titers. Six potential adaptive mutations were identified (I599V in E2, R1373Q and M1611T in NS3, S2364P and C2441S in NS5A and R2523K in NS5B) and the effect of these mutations on HCV replication and infectious particle production was investigated. This cell culture adapted virus enabled us to efficiently infect primary human hepatocytes, as demonstrated using the RFP-NLS-IPS reporter protein and intracellular HCV RNA quantification. However, the induction of a strong type III interferon response in these cells was responsible for HCV inhibition. The disruption of this innate immune response led to a strong infection enhancement and permitted the detection of viral protein expression by western blotting as well as progeny virus production. This cell culture adapted virus also enabled us to easily compare the permissivity of seven hepatoma cell lines. In particular, we demonstrated that HuH-7, HepG2-CD81, PLC/PRF/5 and Hep3B cells were permissive to HCV entry, replication and secretion even if the efficiency was very low in PLC/PRF/5 and Hep3B cells. In contrast, we did not observe any infection of SNU-182, SNU-398 and SNU-449 hepatoma cells. Using iodixanol density gradients, we also demonstrated that the density profiles of HCV particles produced by PLC/PRF/5 and Hep3B cells were different from that of HuH-7 and HepG2-CD81 derived virions. These results will help the development of a physiologically relevant culture system for HCV patient isolates.

## Introduction

Hepatitis C virus (HCV) is a single stranded positive RNA virus that causes serious liver diseases in humans [1]. More than 170 million people worldwide are chronically infected with HCV and are at risk to develop cirrhosis and hepatocellular carcinoma [1]. This virus is a small enveloped virus that belongs to the *Hepacivirus* genus in the *Flaviviridae* family. It contains seven major genotypes and a large number of subtypes [1]. The mechanisms of the HCV life cycle in the liver of infected individuals are only partially understood because of the restricted tropism to humans and chimpanzees and since it has not yet been possible to efficiently infect normal human hepatocytes *in vitro* with serum derived HCV isolates. Thus, the establishment of robust and reliable cell culture systems allowing the study of the whole HCV life cycle is essential to decipher the mechanisms responsible for permissivity to HCV.

A major breakthrough was achieved in HCV field in 2005 thanks to the cloning of a genotype 2a HCV isolate from a Japanese patient with fulminant hepatitis (JFH1 strain) [2]. This genome efficiently replicates in hepatocellular carcinoma HuH-7 cells and its derivatives and enables the production of HCV virions in cell culture (HCVcc) that are infectious to HuH-7 derived cells, chimpanzees, and mice containing human hepatocyte grafts [3]–[6]. Intra- and inter-genotypic chimeras derived from the JFH1 isolate have also been constructed, which has partially allowed for the study of dissimilarities between different genotypes and subtypes [7]. In addition, several adaptive mutations in HCVcc genomes have been reported, which now allow titers to reach up to 10^8^ median tissue culture infective dose (TCID_50_)/mL (for review see [8]). JFH1-based genomes have now been used extensively to dissect the HCV life cycle, however, the question of whether this unusual clone is in fact the “real” virus remains [9]. Differences have been reported between serum derived HCV and HCVcc. For instance, HCV grown *in* *vivo* has a lower buoyant density than HCV grown *in vitro* [6], [10]. Surprisingly, the spectrum of cells permissive to HCVcc infection *in vitro* is principally restricted to HuH-7 derived cells. In addition, the infection of primary human hepatocytes (PHHs) with HCV derived from patient sera or produced in cell culture has proven to be a challenging task. To date, only one group reported robust infection of PHHs with HCVcc [11] while several groups tried to add non-parenchymal feeder cells, as mixed or micropatterned cultures, to stabilize hepatic functions and promote HCVcc infection [12]–[14].

Significant progress has been made in the HCV field, but many challenges still remain [9]. The development of efficient culture systems for the range of viral genotypes still remains an important goal, as it may facilitate the comprehension of the phenotypic differences between clinical isolates and the discovery of broad effective treatments. Similarly, the ability to study the virus in more physiologically relevant environments may yield insights into pathogenesis and persistence. In this study, we performed successive infections of HuH-7 cells with JFH1 derived HCV and obtained a virus able to produce up to 4×10^9^ ffu/mL. This adapted virus enabled us to efficiently infect PHHs and to easily compare the permissivity of several hepatoma cell lines to HCV infection.

## Materials and Methods

### Ethics Statement

The “Biobanque de Picardie” is an internationally recognized ISO 9001 and NF S 96–900 certified Biological Resource Center that pursues its activities according to French laws and regulatory requirements. The French Ministry of Research and Higher Education delivered the authorization N°AC-2010-1165 to collect hepatic resections from the digestive surgery department and then to isolate, store and deliver the PHHs used in this study.

### Cell Culture

HuH-7 (RCB1366) [15], PLC/PRF/5 (CRL-8024) [16], Hep3B (HB-8064) [17], a clone of HepG2 (HB-8065) stably expressing CD81 (HepG2-CD81; S. Belouzard *et al.*, unpublished data), SNU-182 (CRL-2235) [18], SNU-398 (CRL-2233) [18] and SNU-449 (CRL-2234) [18] human hepatocellular carcinoma cell lines as well as Caco-2 human colon adenocarcinoma (HTB-37) [19] and Cos-7 (CRL-1651) African green monkey fibroblast kidney cells [20] were grown at 37°C, 5% CO_2_ in Dulbecco’s Modified Essential Medium (DMEM, Jacques Boy) supplemented with 10% fetal bovine serum.

### Isolation and Culture of PHHs

Liver tissues were obtained from adult patients undergoing partial hepatectomy in Amiens Hospital for the therapy of metastases or benign tumors. These donors were seronegative for HCV, hepatitis B virus, and human immunodeficiency virus. Hepatocytes were isolated from encapsulated liver samples by a 2-step perfusion technique [21], at the “Biobanque de Picardie”. After isolation, hepatocytes were seeded in DMEM supplemented with 10% fetal bovine serum at a density of 2×10^5^ viable cells/cm^2^ onto plates that had been precoated with a solution of type I Rat tail collagen, as recommended by the manufacturer (BD Biosciences). The following day, the medium was replaced with fresh Hepatocyte Culture Medium (Lonza). Alternatively, PHHs were purchased from Biopredic International and cultured in Hepatocyte Culture Medium (Lonza).

### Drugs and Antibodies

2′-C-methylcytidine (2′CMC) and Pyridone-6 (py6) were purchased from Calbiochem. JS-81 anti-CD81 monoclonal antibody (MAb) was purchased from BD Biosciences. Anti-HCV MAbs A4 (anti-E1) [22] and 3/11 (anti-E2; kindly provided by J. McKeating, University of Birmingham, United Kingdom) [23], were produced *in vitro* by using a MiniPerm apparatus (Heraeus), as recommended by the manufacturer. For neutralization assays, the 3/11 MAb was purified using the Pierce Protein G plus Agarose, as recommended by the manufacturer (Pierce).

### Generation of Lentivirus Pseudoparticles and Transductions

Lentivirus pseudoparticles were generated by co-transfection of 293T (CRL-11268) cells with TRIP-RFP-NLS-IPS or TRIP-EGFP-IPS (kindly provided by C.M. Rice), HIV gag-pol, and vesicular stomatitis virus envelope protein G (VSV-G) encoding plasmids, as previously described [24]. HuH-7, HepG2-CD81, Hep3B, PLC/PRF/5, SNU-182, SNU-398, SNU-449, Cos-7 and Caco-2 cells were transduced by overnight incubation with lentivirus pseudoparticles at 37°C to obtain cell lines stably expressing the reporter protein RFP-NLS-IPS or EGFP-IPS. Two days post-seeding, PHHs were transduced by overnight incubation with lentivirus pseudoparticles at 37°C to monitor subsequent HCV infection.

### HCV Virus

We used a plasmid encoding JFH1-CS-A4 genome, a modified version of the full-length JFH1 genome (genotype 2a; GenBank access number AB237837; kindly provided by T. Wakita), which contains mutations leading to amino acids changes F172C and P173S at the C-terminus of the Core protein. These two mutations have been shown to increase the viral titers [25]. In this construct, the N-terminal E1 sequence encoding residues ^196^TSSSYMVTNDC has also been modified to reconstitute the A4 epitope (SSGLYHVTNDC) [22], as previously described [26]. Additionally, we used the plasmid encoding JFH1-CS-A4-Rluc genome, which contains a *Renilla* Luciferase reporter gene and has been described previously [27]. Potential adaptive mutations were introduced in this plasmid using the “QuickChange II XL Site Directed Mutagenesis Kit” (Agilent Technologies). The nucleotide sequences of each mutant were verified. Replication and infectivity were assessed by measuring *Renilla* Luciferase activities using the *Renilla* Luciferase Assay System (Promega) and a Berthold CentroXS3 LB 960 luminometer.

### Production and Adaptation of HCV in Cell Culture

HCV RNA was produced by *in vitro* transcription of the plasmid encoding JFH1-CS-A4 genome and electroporated into HuH-7 cells, as previously described [27]. Supernatant of electroporated cells was recovered ten days after electroporation and filtered through a 0.45-µm-pore-sized membrane (designated as i0 stock). This stock was used to perform 24 successive infections of HuH-7-RFP-NLS-IPS by collecting supernatant when 100% of the cells were infected (designated as i1 to i24 stocks), filtering and transferring virus containing supernatants onto naive cells. Viral supernatants were aliquoted and stored at −80°C.

### Quantitative Reverse-Transcription Polymerase Chain Reaction (RT-qPCR)

HCV RNA in supernatants and total cellular RNA were extracted using the QIAamp viral RNA mini kit (Qiagen) and the RNeasy kit (Qiagen), respectively. cDNAs were synthesized using High Capacity cDNA Reverse Transcription kit and random hexamers, as described by the manufacturer (Applied Biosystems). Amplifications were done with the TaqMan Universal PCR master Mix on an ABI 7900HT Sequence Detection System (Applied Biosystems) using primer and probe sets for HCV RNA [28], IL-28A/B, IL-29, IFN-β and glyceraldehyde 3-phosphate dehydrogenase (GAPDH) (TaqMan Gene Expression Assay, Applied Biosystems). Known copy numbers of *in vitro* transcribed HCV RNA were extracted using the QIAamp viral RNA mini kit (Qiagen) and used to perform a standard curve for the quantification of extracellular HCV RNA. Differential IL-28A/B, IL-29 and IFN-β gene expressions were determined by the ΔΔCt method using GAPDH as endogenous control.

For quantification of miR-122 expression, total RNA was extracted from confluent cells using *mir*Vana™ miRNA Isolation Kit according to the manufacturer’s recommendations (Life Technologies). TaqMan® Small RNA Assays (Life Technologies) was used to performed reverse transcription and real-time quantitative PCR for miR-122-5p (assay ID: 002245) and RNU6B (U6, assay ID: 001093). We utilized the ΔΔCt quantification method, using RNU6B as the endogenous control and the HuH-7 cells as calibrator.

### Quantification of HCV Core Protein

HCV Core was quantified by a fully automated chemiluminescent microparticle immunoassay according to manufacturer’s instructions (Architect HCVAg, Abbott, Germany) [29].

### Cell Viability Assay

HuH-7-RFP-NLS-IPS cells were infected at different multiplicities of infection (MOI). Virus that had been inactivated at 60°C for 30 min was used as a control. 72 h post-infection the viability was measured using the CellTiter-glo® luminescent/Cell viability assay, as recommended by the manufacturer (Promega).

### FFU and TCID_50_ Infectivity Assays

Virus containing supernatants were used to infect naive HuH-7, HepG2-CD81, Hep3B or PLC/PRF/5 cells stably expressing the RFP-NLS-IPS reporter protein. Infected cells were then fixed with paraformaldehyde (4%) at 48 h or 72 h for FFU or TCID_50_ assays, respectively, and checked for fluorescence translocation in the nucleus.

### Cell-to-cell Transmission Assay

6×10^4^ naive HuH-7-RFP-NLS-IPS cells (acceptor cells) were seeded in a 48-well plate, with 300 HuH-7-EGFP-IPS cells, infected with either i0 or i24 (donor cells). Cultures were treated with 50 µg/mL of the 3/11 anti-E2 neutralizing MAb in order to prevent cell-free infection [30]. One day later, cells were fixed with paraformaldehyde and the mean number of HCV infected acceptor cells/focus was determined in 140 separate foci.

### Sequencing of Adaptive Mutations

HCV RNA in supernatants was extracted using the QIAamp viral RNA mini kit (Qiagen). HCV genome sequence was determined by directly sequencing overlapping purified RT-PCR fragments spanning the entire open reading frame, with the BigDye Terminator v1.1 kit (Applied Biosystems). The sequence of primers used for RT-PCR and sequencing reactions are available on request.

### Buoyant Density Iodixanol Gradient Ultracentrifugation

HuH-7, HepG2-CD81, Hep3B and PLC/PRF/5 were electroporated with *in vitro* transcribed RNA of the JFH1-CS-A4-RLuc genome containing mutations R1373Q/C2441S. The supernatants from these cells were recovered six days post-electroporation and 2.5 mL aliquots were overlaid on Iodixanol gradients formed by equal volume (1.5 mL) steps of 10, 15, 20, 25, 30, 35, 40, 45 and 50% (weight/volume) OptiPrep (Sigma) solutions in gradient buffer (150 mM NaCl, 20 mM Hepes/NaOH [pH 7.6], 0.02% bovine serum albumin). Equilibrium was reached by ultracentrifugation for 24 h at 130 000 g in an SW32.1 Ti rotor at 4°C in a Beckman OPTIMA L-100 K BioSafe ultracentrifuge. Sixteen fractions (1 mL) were collected from the top and probed for the HCV RNA level and infectivity measure (assessed by measuring *Renilla* Luciferase activities). The density of the fractions was determined by measuring the mass of each fraction.

## Results

### Generation of Cell Culture Adapted HCV

To study HCV in a more physiologically relevant environment, we aimed at efficiently infecting PHHs with wild-type JFH1. After several unsuccessful attempts, we reasoned that this might be due in part to the relatively low titer obtained with this virus. However, several laboratories have demonstrated that the appearance of adaptive mutations in wild-type JFH1 or derived chimeric viruses, following successive infections of HuH-7 derived cells (up to 13 infections) and/or serial passages of infected cells (up to 6 months) (see [8]). This process has enabled viral titers to reach up to 10^8^ TCID_50_/mL of HCVcc [31]. Thus, in order to increase the chance of PHH infection, we decided to select a virus able to produce high amount of infectious particles by adapting JFH1-CS-A4 to cell culture. This virus contains mutations in the Core protein which have been shown to increase the viral titers (F172C and P173S) [25], and the epitope of A4 MAb in E1 [27]. As previously described, during persistent HCV infection in cell culture, coevolutionary events favouring survival of both virus and host cell take place [32]. For this reason, we chose to perform successive infections, which would be more likely to select a virus with a high capacity of infectious particle secretion. HuH-7 cells were electroporated with JFH1-CS-A4 RNA and the supernatant of the electroporated cells was recovered ten days post-electroporation (supernatant recovered after 0 infection, denoted i0). This supernatant was used to perform successive infections of HuH-7 cells transduced with the reporter protein RFP-NLS-IPS (HuH-7-RFP-NLS-IPS), which enabled us to directly monitor virus spread [24]. This fluorescence-based live cell reporter is composed of a red fluorescent protein (RFP), an SV40 nuclear localization sequence (NLS), and a C-terminal mitochondrial-targeting domain (IPS) derived from the IFN-β promoter stimulator 1 protein (IPS-1), a known cellular substrate for the HCV NS3-4A protease. In HCV infected cells, RFP-NLS-IPS processing by NS3-4A results in translocation of the cleavage product, RFP-NLS, from the mitochondria to the nucleus. Each time cells were 100% infected, we recovered the supernatant (supernatants recovered after “n” infection, denoted i1 to i24) and used it to infect naive HuH-7-RFP-NLS-IPS cells. It has to be noted that for i0, i1 and i2, cells have had to be subcultured before recovering progeny virus, whereas supernatants were directly recovered 3–4 days post-infection for the other cycles of infection.

### Higher Fitness of Cell Culture Adapted HCV

**Figure 1:**
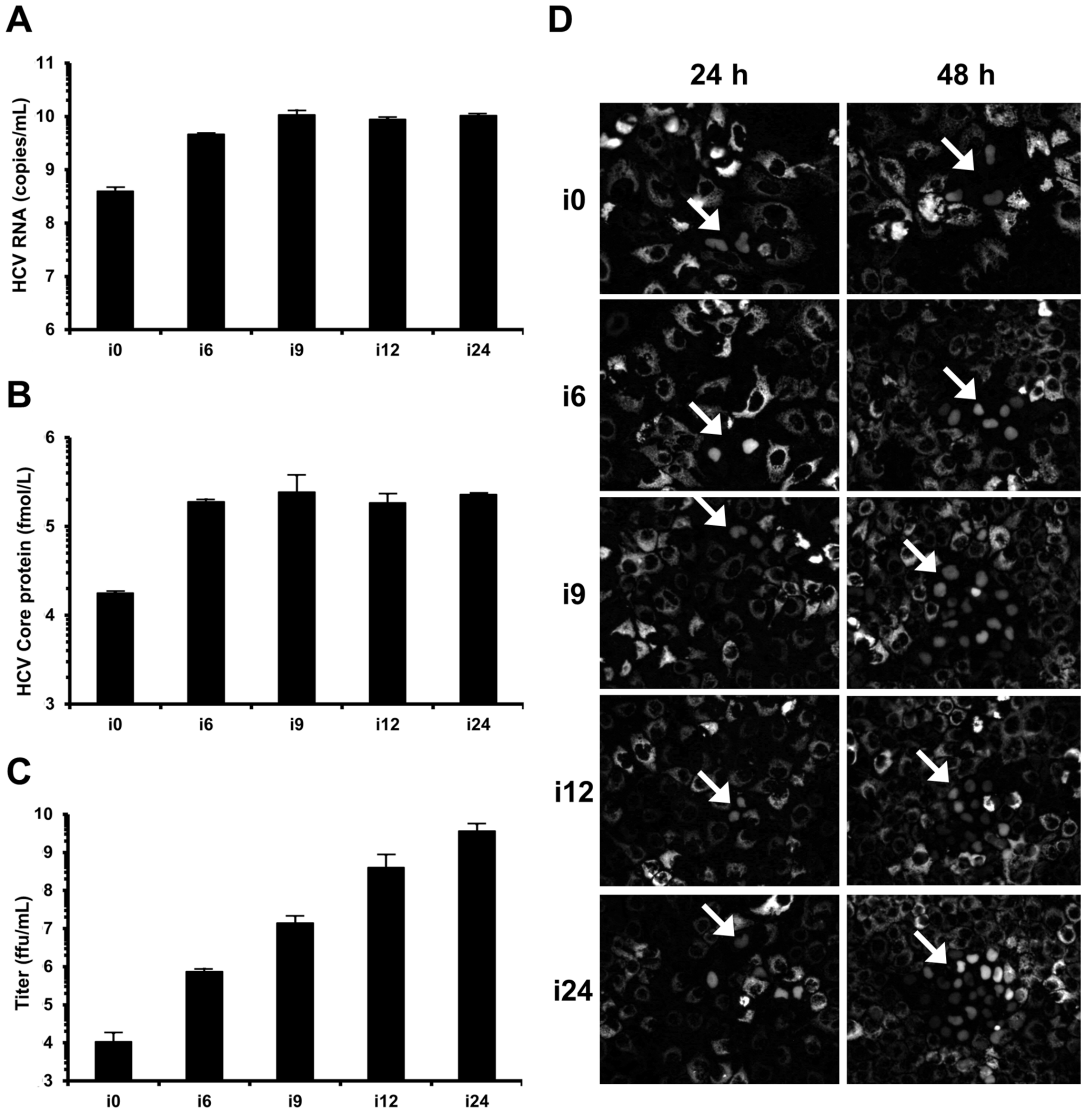
Increase of HCV titers after successive infections. HuH-7 cells were electroporated in the presence of JFH1-CS-A4 RNA. Ten days later, the supernatant of electroporated cells was recovered (denoted supernatant i0) and used to perform successive infections in HuH-7-RFP-NLS-IPS. Each time the cells were 100% infected, the supernatant was recovered (supernatants recovered after “n” infection, denoted i1 to i24) and used to infect naive HuH-7-RFP-NLS-IPS cells. (**A**, **B**) The amount of HCV RNA (**A**) and Core protein (**B**) were quantified in these supernatants by RT-qPCR and fully automated chemiluminescent microparticle immunoassay, respectively. Results are expressed as HCV RNA copies/mL and fmol/L of HCV Core protein, respectively, and are reported as the mean ± S.D. of duplicate and triplicate measurements, respectively. (**C**) Viral titers were determined by ffu assay for i0, i6, i9, i12 and i24. Results are expressed as ffu/mL and are reported as the mean ± S.D. of three independent experiments. (**D**) HuH-7-RFP-NLS-IPS cells were inoculated with the different supernatants at low MOI. Foci of infected cells, identified by translocation of the cleavage product RFP-NLS to the nucleus, were visualized at 24 and 48 h. Images are representative of three independent experiments.

To monitor the adaptation of JFH1-CS-A4, we quantified the amount of HCV RNA and Core protein secreted by infected HuH-7-RFP-NLS-IPS cells in i0, i6, i9, i12 or i24. As shown in Figure 1A, we observed an increase from 4.0×10^8^ to 1.1×10^10^ copies/mL of HCV RNA in i0 and i9 respectively and then a plateau (1.0×10^10^ copies/mL in i24). Similar results were obtained after the quantification of the Core protein in these supernatants (1.8×10^4^, 2.4×10^5^ and 2.3×10^5^ fmol/L of HCV Core protein in i0, i9 and i24, respectively; Figure 1B). These results suggest that physical viral particle secretion increased after the first six successive infections and then reached a plateau.

To further characterize the adapted virus population obtained in these supernatants, we compared infectious viral titers after infection of HuH-7-RFP-NLS-IPS cells. As illustrated in Figure 1C, we observed a gradual increase of the viral titer from 1.1×10^4^ ffu/mL in i0 to 3.7×10^9^ ffu/mL in i24. The increase of physical viral particle secretion may be responsible for the infectious titer enhancement in i6 and i9 compared to i0. Furthermore, it is likely that adaptive mutations which improve viral particle specific infectivity led to the viral titer increase observed in i12 and i24.

When looking at individual foci, we also observed that the virus present in i24 spreads much faster in cell culture than that in i0. Indeed, 24 h after infection, foci were composed of 2-3 infected cells independently of the virus containing supernatant (Figure 1D). In contrast, the day after, the foci observed after inoculation with i24 were almost 10-fold larger than those obtained with i0 (approximately 30 infected cells/focus versus 3 infected cells/focus, respectively). These results suggest that during the 24-successive infections, adaptive mutations improving viral fitness were selected.

**Figure 2:**
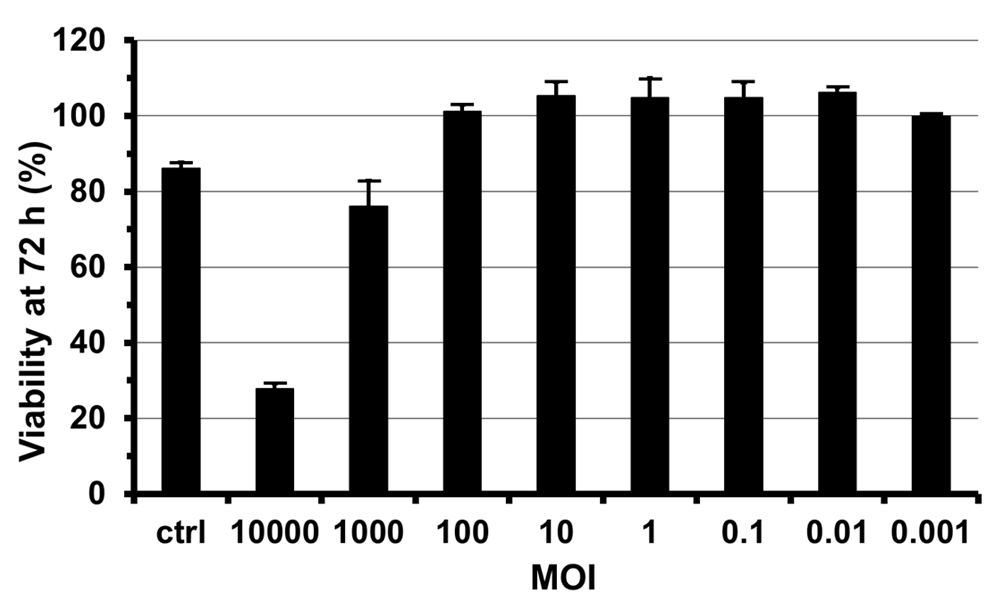
Cytopathic effects induced by cell culture adapted HCV. HuH-7-RFP-NLS-IPS cells were infected with i24 at different MOIs. Non-diluted virus that had been inactivated at 60°C for 30 min was used as control (ctrl). Infected cell viability was evaluated 3 days after infection. The results are expressed as percentages of viability compared to non-infected cells and are reported as means ± S.D. of three independent experiments.

Consistently with previous work [31], we noticed that the adapted virus showed cytopathic effects at high MOI. Specifically, relative to a mock infection, 76 and 28% of HuH-7-RFP-NLS-IPS cells were viable after 3 days of infection at a MOI of 1000 and 10000, respectively, although these MOIs are not physiologically relevant (Figure 2). In contrast, no cytopathic effect was observed 3 days after infection with non-diluted inactivated virus or using MOI of 100 or less.

### Viral Entry of Cell Culture Adapted HCV

**Figure 3:**
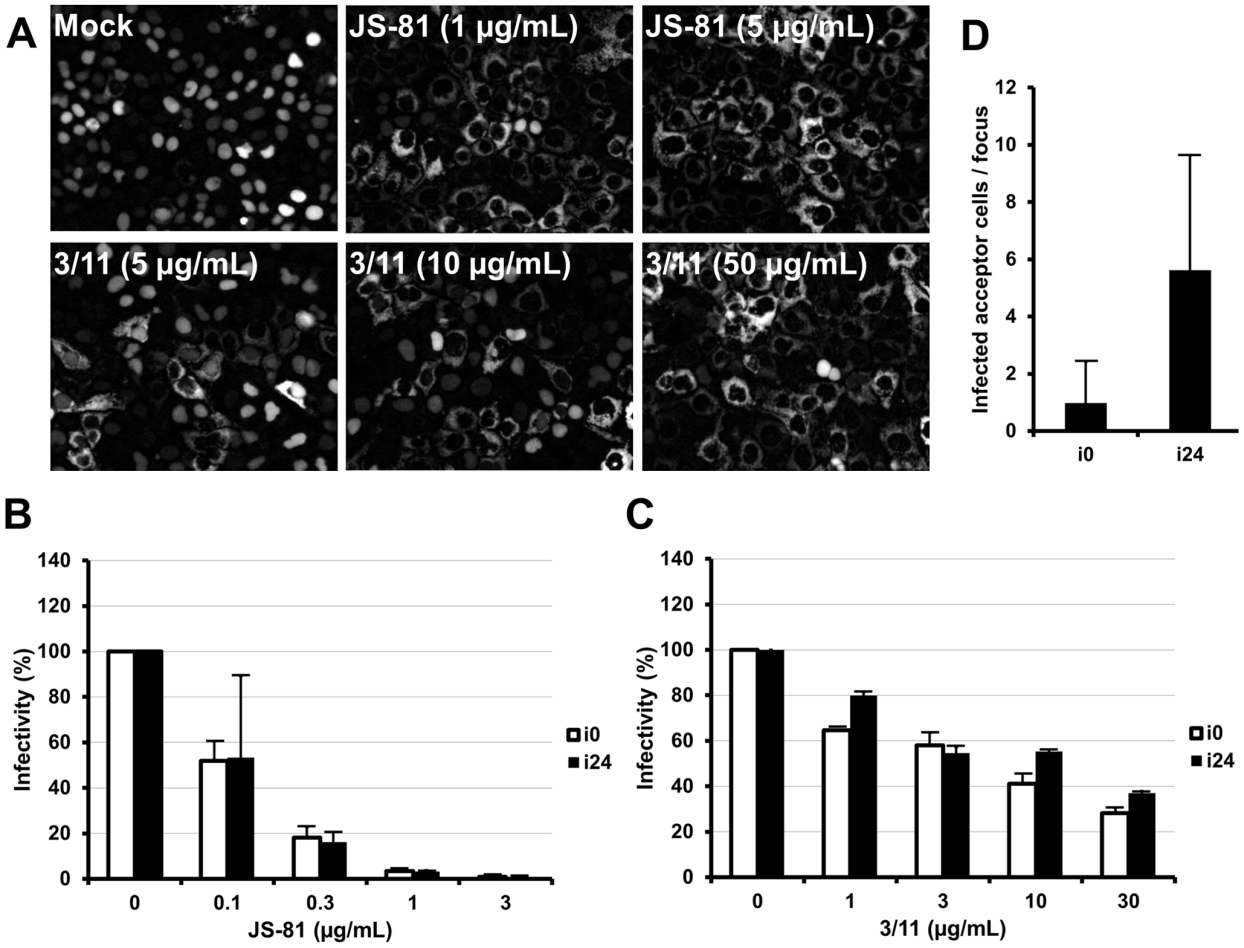
Viral entry of cell culture adapted HCV. (**A**, **B**, **C**) Neutralization of cell culture adapted HCV with 3/11 and JS-81 MAbs. HuH-7-RFP-NLS-IPS cells were infected with i0 or i24 in the absence (Mock) or the presence of 3/11 anti-E2 or JS-81 anti-CD81 MAbs, at the indicated concentration. (**A**) Images taken 48 h after infection with i24 are representative of three independent experiments. (**B**, **C**) Intracellular HCV RNA was quantified 48 h after infection. Results are expressed as percentages of infectivity relative to infectivity in the absence of antibodies and are reported as the means ± S.D. of two independent experiments. (**D**) Cell-to-cell transmission of cell culture adapted HCV. Naive HuH-7-RFP-NLS-IPS cells (acceptor cells) were seeded with HuH-7-EGFP-IPS cells, infected with either i0 or i24 (donor cells). Cultures were treated with 50 µg/mL of the 3/11 anti-E2 neutralizing MAb to prevent cell-free infection. The results are expressed as the mean number of HCV infected acceptor cells/focus ± S.D., determined in 140 separate foci, 24 h post-seeding.

To confirm that our cell culture adapted HCV uses the classically described HCV entry pathway [33], we investigated whether it was neutralized by the 3/11 and the JS-81 MAbs, which recognize the viral E2 envelope protein and the cellular receptor CD81 respectively. As shown in Figure 3A, we noticed that the infection of HuH-7 cells with i24 was dose-dependently neutralized by the MAbs 3/11 and JS-81. These results were confirmed by the quantification of intracellular HCV RNA 48 h post-infection (Figure 3B and 3C). Furthermore, the cell culture adapted and the parental virus were similarly inhibited by both antibodies. Collectively, these results demonstrate that both MAbs neutralize infection in a dose-dependent manner and indicate that the entry of our cell culture adapted HCV occurred in an E2- and CD81-dependent way.

In addition to cell-free virus infection, HCV has been shown to spread efficiently from one infected cell to a neighboring one [34]. This alternative transmission route is resistant to anti-E2 neutralizing antibodies, thus could be important *in vivo*. For this reason, we evaluated the efficiency of cell-to-cell transmission of our cell culture adapted HCV compared to the parental virus. To this end, HuH-7-EGFP-IPS cells (donor), infected with either i0 or i24, were co-cultured with naive HuH-7-RFP-NLS-IPS cells (acceptor) in the presence of 50 µg/mL of the 3/11 neutralizing MAb, in order to prevent cell-free infection, as previously described [30]. One day after seeding, the number of HCV infected acceptor cells per focus was determined for each condition. As shown in Figure 3D, we observed that the efficiency of cell-to-cell transmission was higher for the cell culture adapted HCV than the parental virus (5.6±4.0 and 1.0±1.5 HCV infected acceptor cells/focus, respectively).

### Identification and Characterization of Adaptive Mutations

**Figure 4:**
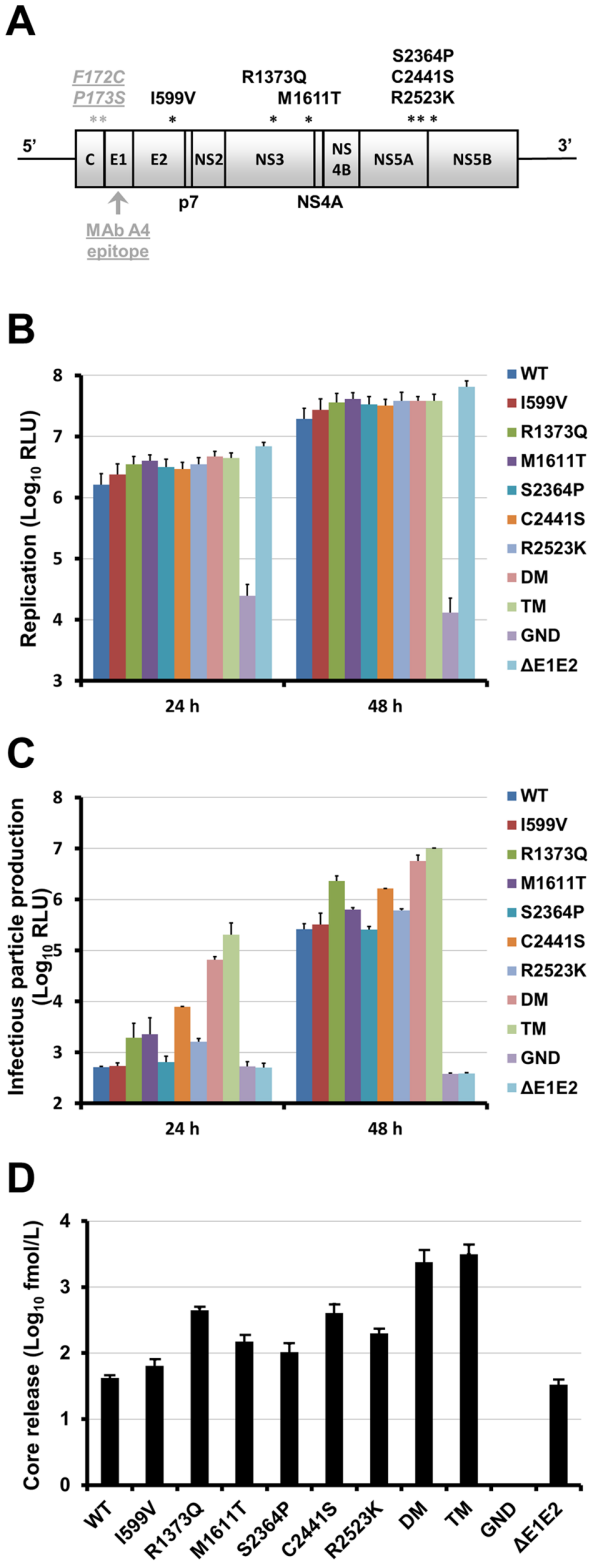
Identification and characterization of potential adaptive mutations. (**A**) Positions of conserved mutations found in the adapted virus on JFH1 open reading frame schematic diagram. The originally introduced amino acid changes F172C and P173S in Core and A4 MAb epitope in E1 are indicated in underlined gray type. Mutations identified at the end of the selection are indicated in black type. (**B**, **C**, **D**) Effect of the potential adaptive mutations on viral genome replication, infectious virus production and HCVcc assembly/secretion. HuH-7-RFP-NLS-IPS cells were transfected with JFH1-CS-A4-RLuc RNA (WT) or mutated HCV genomes (I599V, R1373Q, M1611T, S2364P, C2441S, R2523K, R1373Q/C2441S (DM for double mutant) or R1373Q/M1611T/C2441S (TM for triple mutant)). An assembly-deficient virus (ΔE1E2) and a replication-defective virus (GND) were used as controls. (**B**) Replication was assessed at 4, 24 and 48 h by measuring *Renilla* Luciferase activities in transfected cells. Results are expressed as relative light units (RLU) normalized at 4 h and are reported as the means ± S.D. of two independent experiments. (**C**) The supernatant of transfected cells were recovered at 24 and 48 h and incubated for 3 h with naive HuH-7-RFP-NLS-IPS cells. Luciferase assays were performed on infected cells at 72 h post-infection. Results are expressed as RLU and are reported as the means ± S.D. of two independent experiments. (**D**) HCV core release was quantified in the supernatants recovered 48 h post-transfection. Results are expressed as Core fmol/L and are reported as the means ± S.D. of two independent experiments.

The infectivity difference between i0 and i24 suggested that infectious virions containing adaptive mutations accumulated over time. To identify the mutations responsible for the enhanced virus production, viral RNA from i24 were isolated, amplified by RT-PCR and sequenced. We determined that in addition to the originally introduced amino acid changes F172C and P173S in Core and A4 MAb epitope in E1, the adapted virus contained six non-synonymous mutations compared to the wild-type JFH1 (Figure 4A). One mutation was located in the E2 envelope glycoprotein (I599V) and five were found in the non-structural proteins NS3, NS5A and NS5B (R1373Q and M1611T in NS3, S2364P and C2441S in NS5A, R2523K in NS5B).

To study the role of these mutations on the virus fitness, we introduced them into the JFH1-CS-A4-RLuc genome which contains a *Renilla* Luciferase reporter gene and has been previously described [27]. Interestingly, mutations R1373Q, M1611T and C2441S have already been described by several groups [7], [8], [35]–[38]. Thus, we decided to study two combinations of these mutations, R1373Q/C2441S (DM for double-mutant) and R1373Q/M1611T/C2441S (TM for triple-mutant). A replication-defective virus carrying a mutation in the NS5B GDD motif (GND), and a variant genome carrying a large in-frame deletion in the E1E2 coding region known to inactivate viral particle release (ΔE1E2), were used as negative controls for replication and assembly, respectively [3].

We first assessed the impact of the mutations on HCV RNA replication. As shown in Figure 4B, Luciferase activities in electroporated cells after 24 h and 48 h were slightly increased for the mutants compared to the JFH1-CS-A4-RLuc genome (WT), suggesting that the mutations do not affect the genomic replication of these viruses (between 1.4- and 2.1-fold WT at 48 h). As previously described, the shortened ΔE1E2 genome also replicated slightly faster [39].

To further characterize our mutants, infectious virus production was measured at 4, 24 and 48 h post-electroporation for each mutant and compared to that of the WT. Supernatants of cells transfected with the GND and ΔE1E2 mutants were used as negative controls to determine the background level of Luciferase activity. As shown in Figure 4C, the production of infectious virus for I599V and S2364P mutants was very close to that of the WT. In addition, we observed a slight increase of infectious virus production for mutants M1611T and R2523K (4.4- or 2.5-fold WT for M1611T and 3.2- or 2.3-fold WT for R2523K at 24 or 48 h, respectively) which could be due to the slight increase observed for replication. In contrast, mutations R1373Q and C2441S markedly improved infectious particle production compared to the WT (3.8- or 9.0-fold WT for R1373Q and 15.4- or 6.4-fold WT for C2441S at 24 or 48 h, respectively). Interestingly, these two mutations seemed to have an additive effect since infectious particle production was increased for DM compared to single mutants (130.0- and 22.1-fold WT at 24 and 48 h, respectively). Moreover, addition of the mutation M1611T further increased infectious particle production (399.2- and 38.9-fold WT and then 3.1- and 1.8-fold DM at 24 and 48 h, respectively).

The higher level of infectivity could be the result of improved secretion of viral particles or increased specific infectivity of virions. To distinguish between these two possibilities, we monitored the release of HCV core into the supernatant of electroporated HuH-7 cells using a fully automated chemiluminescent microparticle immunoassay, as previously described [29]. We observed a good correlation when comparing the amount of secreted Core protein (Figure 4D) and infectious particle production (Figure 4C). Indeed, mutants M1611T and R2523K showed a 3.6- and 4.8-fold increase of Core release compared to the WT. The effect of mutations R1373Q and C2441S were more important (10.6- and 9.6-fold WT, respectively). Moreover, the combinations of mutations R1373Q, C2441S and M1611T further enhanced the amount of Core release (56.7- and 75.0-fold WT for DM and TM, respectively). Altogether, these results demonstrate that mutations R1373Q and C2441S are of major importance for the increased fitness of our cell culture adapted virus, through secretion enhancement. They also suggest a beneficial role of mutations M1611T and R2523K on secretion. Whether mutations I599V and S2364P have an additive effect in combination with the other mutations remains to be determined.

### Permissivity of PHHs to Cell Culture Adapted HCV

**Figure 5:**
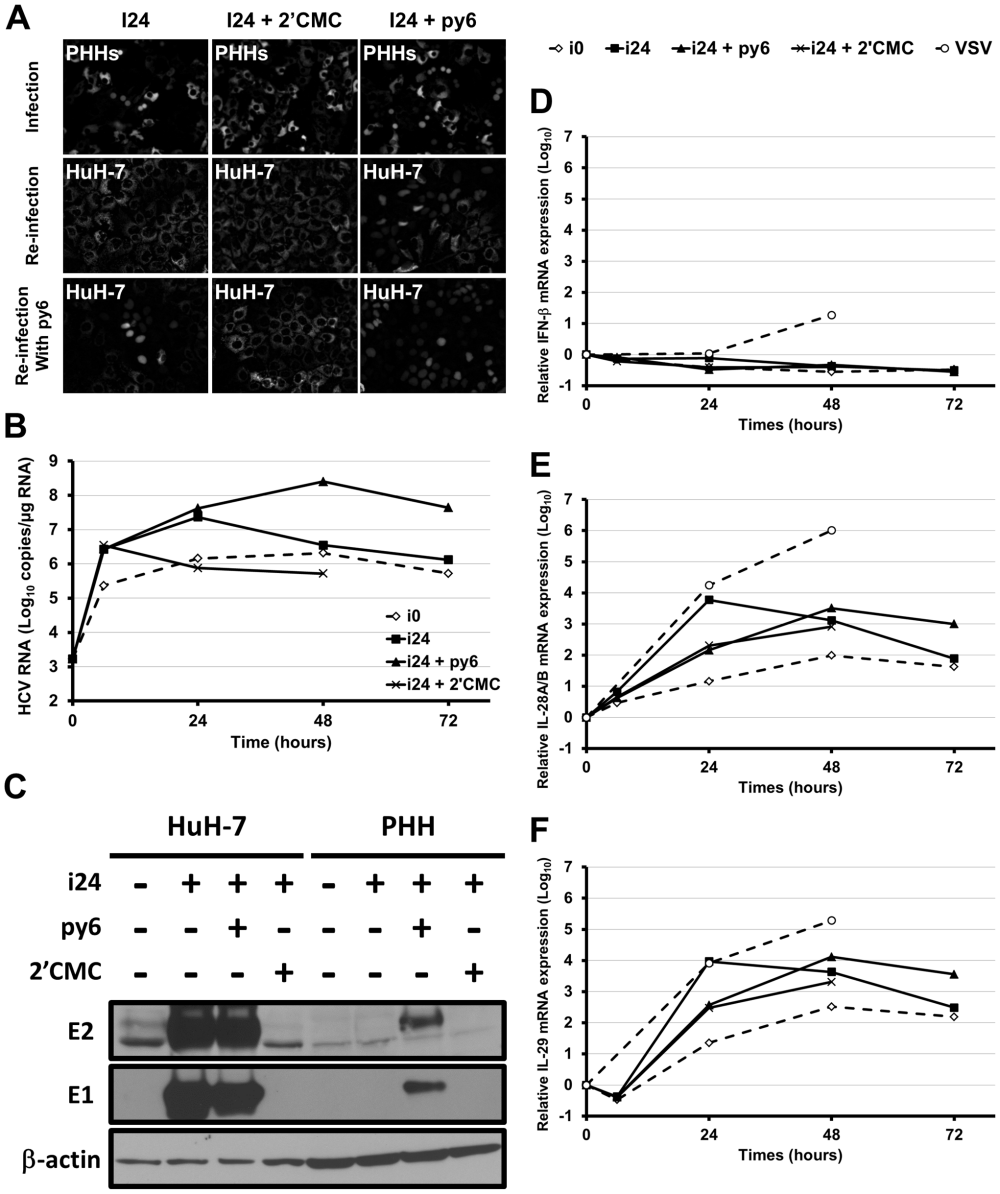
Infection of PHHs with cell culture adapted HCV. PHHs from one representative donor were inoculated for 6 h with non-adapted HCV (i0; MOI = 0.01 HuH-7 infectious units per cell) or i24 (MOI = 1000 HuH-7 infectious units per cell), in the presence or absence of 2′CMC (10 µM) or py6 (500 nM). After inoculation, cells were washed three times with PBS and new media containing the drugs were added and replaced every day. (**A**) Infection of PHHs that had previously been transduced with lentivirus expressing RFP-NLS-IPS, was visualized 48 h post-infection by translocation of the cleavage product RFP-NLS to the nucleus (“Infection” panel). The supernatants of inoculated cells were recovered 48 h post-infection, centrifuged and used to inoculate naive HuH-7-RFP-NLS-IPS in the absence or presence of py6 (“Re-infection” and “Re-infection with py6” panels, respectively) to check the production of progeny virus. Infected HuH-7 cells were visualized 48 h post-infection. (**B**) Intracellular HCV RNA was quantified by RT-qPCR, after inoculation of non-transduced PHHs. Results are expressed as means ± S.D. of duplicates. (**C**) Expression of the viral proteins E1 and E2 was analyzed 48 h post-infection in cell lysates by Western blotting using specific MAbs (A4 [anti-E1], 3/11 [anti-E2]**,** and C4 [anti-β-actin]). HuH-7 cells infected in the same conditions were used as control. (**D**, **E**, **F**) IFN-β, IL-28A/B and IL-29 expression in infected PHHs was determined in duplicate by RT-qPCR. The results are normalized to GAPDH endogenous control and presented as fold-increase over pre-infection levels, using the ΔΔCt method.

The aim of this study was to produce high titer HCVcc able to efficiently infect PHHs. Since our adapted JFH1 achieved 3.7×10^9^ ffu/mL, we investigated whether robust infection of PHHs could be detected with this virus. To this end, we transduced PHHs prepared from different donors with lentivirus expressing RFP-NLS-IPS and inoculated them with i24 at least 24 hours post-transduction. Importantly, we investigated whether expression of interferons was induced following transduction, however we did not evidence such induction which could have had an effect on subsequent HCV infection (data not shown). When inoculating transduced PHHs with the non-adapted JFH1 (i0) only rare positive cells were detected with a few batches of PHHs (data not shown). In contrast, using our cell culture adapted HCV (i24), we observed fluorescence translocation in the nucleus of numerous inoculated PHHs 48 h post-infection for all the preparations tested (more than ten), demonstrating that this virus efficiently infects PHHs and that the genome was at least translated (Figure 5A). In addition, when 2′-C-Methylcytidine (2′CMC), a potent inhibitor of the HCV NS5B RNA polymerase, was added during and after inoculation, the number of fluorescent nuclei was strongly decreased indicating that the genome also replicates in PHHs (Figure 5A). We observed that PHHs were still permissive to HCV infection 20 days post-platting (data not shown). In order to estimate the efficiency of infection, we counted the number of fluorescent nuclei on four pictures, considering that the probability of HCV infection was identical in transduced and non-transduced PHHs. We determined that the efficiency was dependent on the donor however we obtained up to 28% of infected PHHs (40 infected PHHs out of 139).

In order to confirm PHH infection, we quantified intracellular HCV RNA at different time points after inoculation of non-transduced PHHs with i24 (Figure 5B). In the presence of 2′CMC, we observed a decrease of intracellular HCV RNA from 3.5×10^6^ to 7.6×10^5^ copies/µg RNA, between 6 h (the end of the inoculation) and 24 h. In contrast, in the same period, we detected an increase from 2.7×10^6^ to 2.3×10^7^ copies/µg RNA without 2′CMC, confirming that HCV had infected the cells and that replication occurred. Intracellular HCV RNA decreased from 2.3×10^7^ to 3.5×10^6^ between 24 and 48 h. When we used non-adapted virus as a control (i0), we also observed an increase of intracellular HCV RNA (from 2.3×10^5^ to 2.1×10^6^ copies/µg RNA between 6 and 48 h). However, as mentioned above, this amount was not sufficient to clearly visualize infection by RFP-NLS-IPS cleavage in transduced PHHs that were used as control. We also analyzed the expression of viral proteins by western blotting on non-transduced PHH lysates, using HuH-7 cells infected in the same conditions as positive control (Figure 5C). However, whereas E1 and E2 expression was easily detected in infected HuH-7 cells, we did not detect any expression in infected PHHs.

To investigate whether progeny virus was produced from infected PHHs, culture supernatant was recovered two days post-infection, centrifuged and used to inoculate naive HuH-7-RFP-NLS-IPS cells. However, as shown in Figure 5A (see “Re-infection”) we did not detect any infected HuH-7 cells. As previously reported [14], we did not observe virus spreading in the cultured PHHs and in contrast we noticed that the number of cells with fluorescence translocation in the nucleus was strongly decreased 7-days post-infection (data not shown). This suggested that infected cells either cleared the virus or died.

Several studies have recently pointed to strong type III and weak type I IFN responses induced after HCV infection of PHHs [40]–[42]. For this reason we investigated whether innate immunity induced after infection with our cell culture adapted HCV could be responsible for the absence of viral spread and virus clearance. To this end, we quantified by RT-qPCR the expression of IFN-β, IL-28A/B and IL-29 in PHHs infected with HCV or Vesicular Stomatitis Virus (VSV), which was used as a positive control. As shown in Figure 5D, we did not detect any induction of IFN-β expression after infection with non-adapted JFH1 (i0) or our cell culture adapted HCV (i24). In contrast, according to the literature [40]–[42], we observed a strong induction of IL-28A/B (Figure 5E) and IL-29 (Figure 5F) expression after infection with i24. A lower induction was also observed using the non-adapted HCV (i0) or when infecting PHHs with i24 in the presence of 2′CMC.

To confirm that this innate immune response had a negative effect on HCV infection, we infected PHHs with i24 in the presence of the pan-JAK inhibitor pyridone-6 (py6), as previously described [40]. Interestingly, when py6 was added during and after HCV infection, we observed a strong increase of infected PHH number (Figure 5A), suggesting a more efficient viral replication and potential propagation of the virus. In line with this result we observed a 1.8 and 1.5-Log_10_ increase of HCV intracellular RNA at 48 and 72 h, compared to the infection in the absence of py6 (Figure 5B). Furthermore we were able to detect viral protein expression by western blotting (Figure 5C). Noticeably, py6 did not have any effect on HCV infection in HuH-7 cells (Figure 5C). Production of progeny virus was also detected when PHHs were infected in the presence of py6 (Figure 5A, see “Re-infection”). Finally, all these results were accompanied with a delay of IL-28A/B and IL-29 expression induction, probably due to the disruption of positive feedback loops by the py6 [43].

Since IFN-λ secreted by the infected PHHs could inhibit infection of the subsequently inoculated cultures, we added py6 during the inoculation of HuH-7 cells with the supernatants of infected PHHs. As shown in Figure 5A (see “Re-infection with py6”), we observed that the addition of py6 increased infection of HuH-7 cells inoculated with the supernatant of PHHs that had been infected in the presence of py6. More importantly, the addition of py6 during the inoculation of HuH-7 with the supernatant of PHHs that had been infected in the absence of py6 resulted in the detection of a few positive foci. As a negative control, no progeny virus was detected when PHH infection had been performed in the presence of 2′CMC. No production of progeny virus was observed after PHH infection with non-adapted virus even when subsequent inoculation was performed in the presence of py6 (data not shown). These results demonstrate that PHHs infected with our cell culture adapted HCV produced infectious virions and that concomitant secretion of IFN-λ by PHHs inhibits the infection of the subsequently inoculated cultures.

### Permissivity of Hepatoma Cell Lines to Cell Culture Adapted HCV

**Figure 6:**
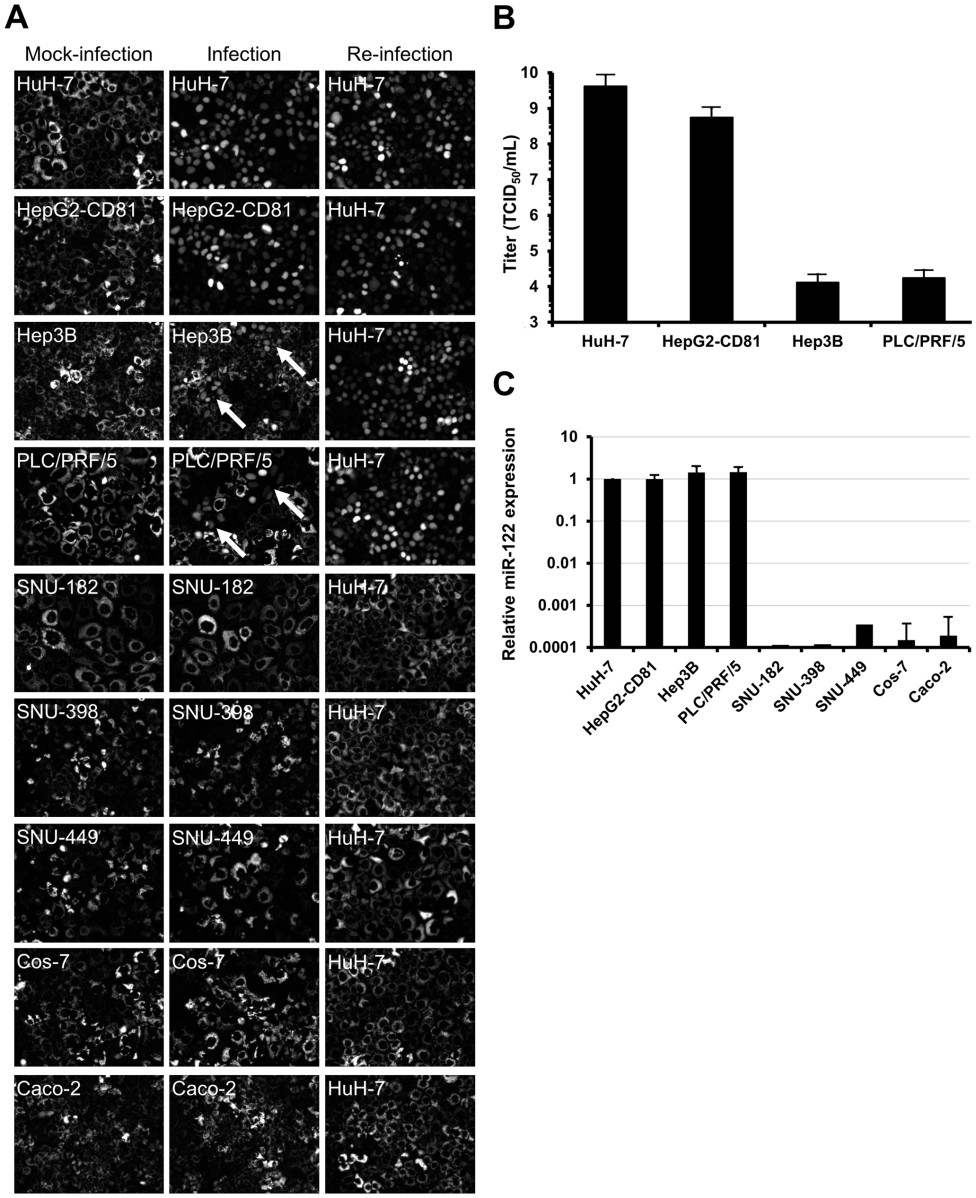
Infection of hepatoma cell lines with cell culture adapted HCV. (**A**) HuH-7, HepG2-CD81, Hep3B, PLC/PRF/5, SNU-182, SNU-398, SNU-449, Cos-7 and Caco-2 cells transduced with lentivirus expressing RFP-NLS-IPS were mock-infected (left) or inoculated with i24 (middle) (MOI = 10000 HuH-7 infectious units per cell). Infected cells, identified by translocation of the cleavage product RFP-NLS to the nucleus, were visualized 48 h post-infection. The supernatants of inoculated cells were recovered 72 h post-infection, centrifuged and used to inoculate naive HuH-7-RFP-NLS-IPS to check the production of progeny virus (right). Images are representative of three independent experiments. (**B**) The permissivity of HuH-7, HepG2-CD81, Hep3B and PLC/PRF/5 cells to the cell culture adapted virus was determined by TCID_50_ assay. The results are expressed as TCID_50_/mL ± S.D. calculated on 8 wells. (**C**) miR-122 expression was determined by RT-qPCR in HuH-7, HepG2-CD81, Hep3B, PLC/PRF/5, SNU-182, SNU-389, SNU-449, Cos-7 and Caco-2 cells. The results, which are representative of four independent experiments, are expressed as relative miR-122 expression using the ΔΔCt method with RNU6B as endogenous control and HuH-7 cells as calibrator.

Discrepant results have recently been published concerning the permissivity of HepG2-CD81, Hep3B and PLC/PRF/5 cells to HCV infection [44]–[47]. We decided to investigate the permissivity of these cells to our cell culture adapted virus and to extend our study using other hepatoma cell lines. We transduced HepG2-CD81, Hep3B, PLC/PRF/5, SNU-182, SNU-398 and SNU-449 with lentivirus expressing RFP-NLS-IPS in order to obtain cell lines stably expressing the reporter protein. Control cell lines were obtained after transduction of Cos-7 and Caco-2, which are of kidney and colon origin, respectively. After inoculation of the transduced cells with i24, we detected RFP-NLS-IPS cleavage in HuH-7, HepG2-CD81, Hep3B and PLC/PRF/5 (Figure 6A, compare left and middle). Specifically, HuH-7 and HepG2-CD81 cells were 100% infected, whereas we observed isolated foci in Hep3B and PLC/PRF/5 cells (indicated by arrows). In contrast, we did not detect any RFP-NLS-IPS cleavage in SNU-182, SNU-398, SNU-449 as well as the Cos-7 and Caco-2 control cells (Figure 6A middle).

In order to compare the permissivity of HuH-7, HepG2-CD81, Hep3B and PLC/PRF/5 cell lines to our adapted virus, we quantified the TCID_50_ for each cell line. As shown in Figure 6B, HepG2-CD81 cells were almost 10-fold less permissive to HCV infection than HuH-7 cells (5.6×10^8^ and 4.2×10^9^ TCID_50_/mL, respectively). Furthermore, we observed a 5 log_10_ difference in the permissivity of Hep3B and PLC/PRF/5 cells compared to HuH-7 cells (1.3×10^4^, 1.8×10^4^ and 4.2×10^9^ TCID_50_/mL, respectively). Several studies have recently described that HepG2-CD81, Hep3B and PLC/PRF/5 cells express low levels of miR-122, a dependency factor to HCV replication [44], [45]. For this reason, we measured the expression of miR-122 in our cells and observed that the expression of this miRNA was similar in HuH-7, HepG2-CD81, Hep3B and PLC/PRF/5, whereas it was undetectable in SNU-182, SNU-398, SNU-449, Caco-2 and Cos-7 (Figure 6C).

To investigate whether progeny virus was produced from infected HepG2-CD81, Hep3B and PLC/PRF/5, the culture supernatants of these cells were recovered, centrifuged and used to inoculate naive HuH-7-RFP-NLS-IPS cells. As shown in Figure 6A (right), 48 h post-infection we observed in each case that 100% of HuH-7-RFP-NLS-IPS cells were infected demonstrating that HepG2-CD81, Hep3B and PLC/PRF/5 are able to produce progeny virus. As a negative control, we did not detect any fluorescence translocation when we put naive HuH-7-RFP-NLS-IPS cells into contact with the supernatant of inoculated SNU-182, SNU-398, SNU-449 as well as the Cos-7 and Caco-2 control cells.

### Profiles of Density of HCVcc Produced in Different Hepatoma Cell Lines

**Figure 7:**
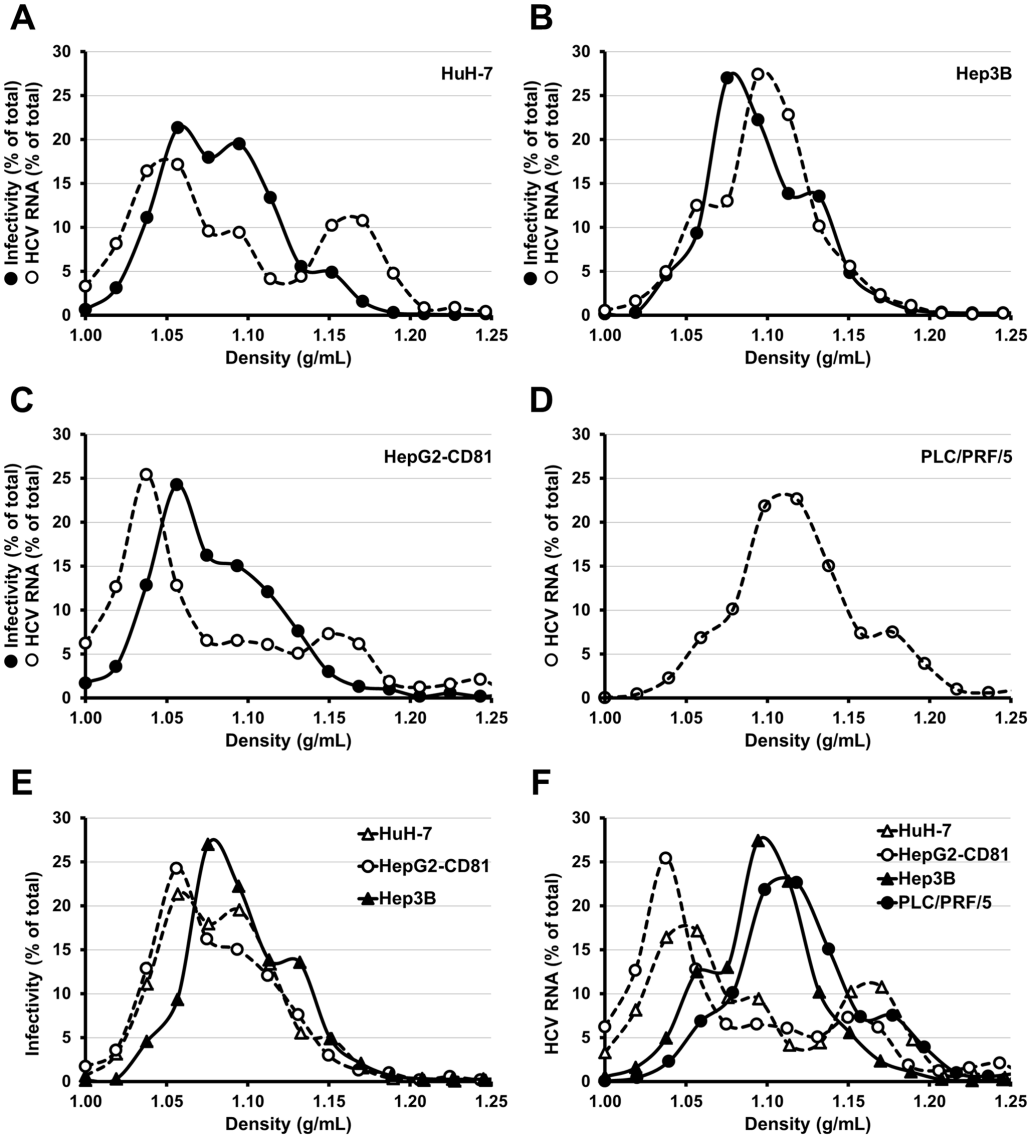
Profiles of density of HCV produced in different hepatoma cell lines. HuH-7 (**A**), Hep3B (**B**), HepG2-CD81 (**C**) and PLC/PRF/5 (**D**) were electroporated with *in vitro* transcribed RNA of the JFH1-CS-A4-RLuc genome containing mutations R1373Q/C2441S. The supernatants of each electroporated cell lines were recovered six days post-electroporation and overlaid on 10 to 50% (weight/volume) iodixanol gradients. After a 24 h ultracentrifugation, sixteen fractions were collected and analyzed for HCV RNA quantity and infectivity on naive HuH-7 cells (assessed by measuring *Renilla* Luciferase activities). The results are expressed as percentages of total infectivity or HCV RNA and are reported as means of two independent experiments.

Serum derived HCV has a lower buoyant density than JFH1 grown *in vitro* in HuH-7 cells [6], [10]. Since HepG2-CD81, Hep3B and PLC/PRF/5 are able to produce progeny virus, we investigated the density profile of virions secreted by these cell lines. We electroporated HuH-7, HepG2-CD81, Hep3B and PLC/PRF/5 with *in vitro* transcribed RNA of a JFH1 derived virus containing a Luciferase reporter gene. Cells were trypsinized three days post-electroporation and the supernatants of each electroporated cell lines were recovered six days post-electroporation. These supernatants were overlaid on 10 to 50% (weight/volume) iodixanol gradients and equilibrium was reached through a 24 h ultracentrifugation. Sixteen fractions were collected and probed for HCV RNA level and infectivity. As shown in Figure 7, the density profile of virions produced by HepG2-CD81 (Figure 7C) was very similar to that of HuH-7 derived virions (Figure 7A), with a major peak around 1.05 g/mL and a minor peak around 1.16 g/mL for HCV RNA as well as a peak at 1.06 g/mL with a shoulder until 1.15 g/mL for infectivity. In contrast, the density profiles of Hep3B (Figure 7B) and PLC/PRF/5 (Figure 7D) derived virions were different. The RNA peaked at a density comprised between 1.09 and 1.12 g/mL and the peak of infectivity was observed around 1.08 g/mL for Hep3B derived virions. Unfortunately, the amount of Luciferase expressing virus produced by PLC/PRF/5 was too low and we could not detect any specific infectivity signal after inoculation of naive HuH-7 cells with the fractions of this gradient. These differences are brought to the fore when comparing infectivity or HCV RNA density profiles of HuH-7, HepG2-CD81, Hep3B, PLC/PRF/5 derived virions on the same graph (Figure 7E and 7F, respectively).

## Discussion

In order to increase the chance of PHH infection, we selected a virus able to produce high amount of infectious particles by adapting a JFH1 derived genome in HuH-7 cells. After twenty-four successive infections, we obtained a virus that reaches titers up to 4×10^9^ ffu/mL. In addition to the F172C and P173S mutations originally introduced in the Core protein and known to increase viral particle secretion [25], we identified six putative adaptive mutations which could be responsible for the viral titer increase (R1373Q and M1611T in NS3, S2364P and C2441S in NS5A, R2523K in NS5B). Our results demonstrate that the mutations R1373Q and C2441S are of major importance for the increased fitness of our cell culture adapted virus and also suggest a beneficial role of the mutations M1611T and R2523K. Interestingly, mutation R1373Q has already been shown to increase the trans-encapsidation of a JFH1 replicon lacking envelope protein genes [38] as well as the production of J8/JFH1 (2b/2a), S52/JFH1 (3a/2a) and DH5/JFH1 (1b/2a) chimeras [7], [8], [35]. Furthermore, several adaptive mutations have been identified in the last amino acids of NS5A, particularly at position 2441 [36], [37], [48]–[51]. Besides, the mutation C2441S has also recently been found in association with the mutation M1611T [37]. In contrast, our data do not point to an essential role of mutations I599V and S2364P, however we cannot exclude that they have an additive effect in combination with the other mutations.

PHH infection with HCV derived from patient sera or produced in cell culture has proven to be a challenging task. Several groups tried to add non-parenchymal feeder cells, as mixed or micropatterned cultures, to stabilize hepatic functions [12]–[14], but the enhancement of HCV infection was limited and to date only one group reported robust infection of PHHs and production of progeny virions by infected PHHs [11]. The generation of a cell culture adapted HCV enabled us to obtain such a model that will permit the study of the HCV viral cycle in a physiologically relevant environment. Using this cell culture system, we observed that induction of a strong type III interferon response in infected PHHs was responsible for HCV inhibition, confirming the results of recent studies [40]–[42]. The disruption of this immune response led to a strong enhancement of HCV infection and progeny virus production. Thus, our model of PHH infection also provides an informative environment for studying the cellular mechanisms that operate to limit viral spread.

Our results also show that HepG2-CD81, Hep3B and PLC/PRF/5 are permissive to HCV entry, replication and assembly. Interestingly, during this work, other studies have been published concerning HepG2-CD81, Hep3B and PLC/PRF/5 permissivity to HCV infection [44]–[47]. Sainz *et al.* demonstrated that HepG2-CD81 and Hep3B cells are permissive to the full JFH1 viral cycle despite a lower efficiency compared to HuH-7 cells [46]. In addition, it has been shown that these cells express low levels of miR-122 and exhibit a significant enhancement of HCV replication after transduction of this miRNA [44], [45], [47]. Conflicting results have been published concerning PLC/PRF/5 permissivity to HCV. Indeed, Kambara *et al.* did not observe any viral replication in these cells regardless of miR-122 expression [44], whereas Sainz *et al.* demonstrated that they are permissive to JFH1 entry and replication, but defective for *de novo* HCVcc production [46]. Some discrepancies have also been published concerning Caco-2 permissivity to HCV [46], [52]. In accordance with Sainz *et al.* [46] and in contrast to Mee *et al.* [52], we did not detect any infection of these cells using our cell culture adapted virus. In contrast to several studies [44], [45], [47], it is important to note that we detected similar levels of miR-122 expression in HuH-7, HepG2-CD81, Hep3B and PLC/PRF/5 and undetectable or very low levels in SNU-182, SNU-398, SNU-449, Caco-2 and Cos-7 cells. This result is not surprising for the clone of HepG2-CD81 we used, since it had been selected for its permissivity to HCV infection. Concerning Hep3B and PLC/PRF/5, it is likely that cells have evolved in different ways from one lab to the other and acquired phenotypic differences, as already observed for HuH-7 cells [53]. Importantly, conflicting results have already been published concerning the relative expression of miR-122 in these different hepatoma cell lines [44], [54]. In addition, it has to be noted that miR-122-independent HCV replication has recently been described [47] **.** We did not observe any infection of SNU-182, SNU-398 and SNU-449 cells with HCVcc. These three cell lines thus provide additional tools to identify new dependency and/or restriction factors to HCV infection.

Differences have been reported between serum derived HCV and HCVcc produced in HuH-7 cells, particularly concerning the density of physical viral particles, which is lower for HCV grown *in* *vivo* [6], [10]. It is now known that HCV assembly is tightly linked to the Very Low Density Lipoprotein (VLDL) assembly process [10]. VLDL biogenesis is envisioned as a two-step process, which requires at least apolipoprotein B (ApoB) and the microsomal triglyceride transfer protein (MTP). Ongoing translation of ApoB concomitant with MTP-mediated lipid transfer forms a neutral lipid core that is converted into a nascent VLDL acquiring exchangeable ApoE and ApoC, also designated VLDL2. This VLDL2 can be released as such or fuse with ApoB-free luminal lipid droplets that are secondary precursors, formed by MTP in the smooth endoplasmic reticulum to give a triglyceride-rich lipoproteins designated VLDL1. Recently, it has been proposed that during HCV assembly, the nucleocapsid as well as E1 and E2 glycoproteins would be inserted into the ApoB-free luminal lipid droplets [10], [55]. Thus, in VLDL competent cells, as found *in vivo*, this precursor could fuse with VLDL2 to form a lipoviroparticle. In contrast, it has been proposed that HCVcc is secreted predominantly as particles lacking ApoB by HuH-7 cells since VLDL1 formation is inefficient [55]. We noticed that the density of viral particles produced by Hep3B and PLC/PRF/5 was different than that of HuH-7 and HepG2-CD81 derived HCV. Our results concerning HepG2-CD81 derived HCV are in agreement with a recent study which demonstrated that HepG2 cells produce infectious virions that are biophysically and biochemically similar to HuH-7 derived virions [56]. Interestingly, Forte *et al.* reported in 1989, that contrary to HepG2 cells, Hep3B and NPLC/PRF/5 cells (a subline of PLC/PRF/5) secreted quantitatively significant amounts of lipoproteins corresponding to the three major density classes of plasma [57]. They also described that like plasma VLDL, Hep3B VLDL contained ApoB, ApoC, and ApoE [57]. For these reasons it would be interesting to compare the assembly process and the biochemical content of HCVcc produced by these different cell lines.

Altogether, our results bring new insights into the amplification of HCV in cell culture. This will help the development of efficient culture systems to study all viral genotypes in more physiologically relevant environments.

## References

[1] Lemon SM, Walker C, Alter MJ, Yi M (2007) Hepatitis C virus. In: Knipe DM, Howley PM, editors. Fields Virology. Philadelphia, Pa: Lippincott Williams & Wilkins. 1253–1304.

[2] Kato T, Date T, Miyamoto M, Furusaka A, Tokushige K, et al (2003) Efficient replication of the genotype 2a hepatitis C virus subgenomic replicon. Gastroenterology 125: 1808–1817. 14724833 10.1053/j.gastro.2003.09.023

[3] Wakita T, Pietschmann T, Kato T, Date T, Miyamoto M, et al (2005) Production of infectious hepatitis C virus in tissue culture from a cloned viral genome. Nat Med 11: 791–796. 15951748 10.1038/nm1268 PMC2918402

[4] Lindenbach BD, Evans MJ, Syder AJ, Wolk B, Tellinghuisen TL, et al (2005) Complete replication of hepatitis C virus in cell culture. Science 309: 623–626. 15947137 10.1126/science.1114016

[5] Zhong J, Gastaminza P, Cheng G, Kapadia S, Kato T, et al (2005) Robust hepatitis C virus infection in vitro. Proc Natl Acad Sci USA 102: 9294–9299. 15939869 10.1073/pnas.0503596102 PMC1166622

[6] Lindenbach BD, Meuleman P, Ploss A, Vanwolleghem T, Syder AJ, et al (2006) Cell culture-grown hepatitis C virus is infectious in vivo and can be recultured in vitro. Proc Natl Acad Sci USA 103: 3805–3809. 16484368 10.1073/pnas.0511218103 PMC1533780

[7] Gottwein JM, Scheel TK, Jensen TB, Lademann JB, Prentoe JC, et al (2009) Development and characterization of hepatitis C virus genotype 1–7 cell culture systems: role of CD81 and scavenger receptor class B type I and effect of antiviral drugs. Hepatology 49: 364–377. 19148942 10.1002/hep.22673

[8] Scheel TK, Gottwein JM, Carlsen TH, Li YP, Jensen TB, et al (2011) Efficient culture adaptation of hepatitis C virus recombinants with genotype-specific core-NS2 by using previously identified mutations. J Virol 85: 2891–2906. 21177811 10.1128/JVI.01605-10 PMC3067958

[9] Murray CL, Rice CM (2011) Turning hepatitis C into a real virus. Annu Rev Microbiol 65: 307–327. 21682640 10.1146/annurev-micro-090110-102954

[10] Bartenschlager R, Penin F, Lohmann V, Andre P (2011) Assembly of infectious hepatitis C virus particles. Trends Microbiol 19: 95–103. 21146993 10.1016/j.tim.2010.11.005

[11] Podevin P, Carpentier A, Pene V, Aoudjehane L, Carriere M, et al (2010) Production of infectious hepatitis C virus in primary cultures of human adult hepatocytes. Gastroenterology 139: 1355–1364. 20600021 10.1053/j.gastro.2010.06.058

[12] Banaudha K, Orenstein JM, Korolnek T, St Laurent GC (2010) Primary hepatocyte culture supports hepatitis C virus replication: a model for infection-associated hepatocarcinogenesis. Hepatology 51: 1922–1932. 20512986 10.1002/hep.23616

[13] Buck M (2008) Direct infection and replication of naturally occurring hepatitis C virus genotypes 1, 2, 3 and 4 in normal human hepatocyte cultures. PLoS One 3: e2660. 18628977 10.1371/journal.pone.0002660 PMC2442186

[14] Ploss A, Khetani SR, Jones CT, Syder AJ, Trehan K, et al (2010) Persistent hepatitis C virus infection in microscale primary human hepatocyte cultures. Proc Natl Acad Sci U S A 107: 3141–3145. 20133632 10.1073/pnas.0915130107 PMC2840339

[15] Nakabayashi H, Taketa K, Miyano K, Yamane T, Sato J (1982) Growth of human hepatoma cells lines with differentiated functions in chemically defined medium. Cancer Res 42: 3858–3863. 6286115

[16] MacNab GM, Alexander JJ, Lecatsas G, Bey EM, Urbanowicz JM (1976) Hepatitis B surface antigen produced by a human hepatoma cell line. Br J Cancer 34: 509–515. 187208 10.1038/bjc.1976.205 PMC2025201

[17] Aden DP, Fogel A, Plotkin S, Damjanov I, Knowles BB (1979) Controlled synthesis of HBsAg in a differentiated human liver carcinoma-derived cell line. Nature 282: 615–616. 233137 10.1038/282615a0

[18] Park JG, Lee JH, Kang MS, Park KJ, Jeon YM, et al (1995) Characterization of cell lines established from human hepatocellular carcinoma. Int J Cancer 62: 276–282. 7543080 10.1002/ijc.2910620308

[19] Fogh J, Fogh JM, Orfeo T (1977) One hundred and twenty-seven cultured human tumor cell lines producing tumors in nude mice. J Natl Cancer Inst 59: 221–226. 327080 10.1093/jnci/59.1.221

[20] Gluzman Y (1981) SV40-transformed simian cells support the replication of early SV40 mutants. Cell 23: 175–182. 6260373 10.1016/0092-8674(81)90282-8

[21] Biron-Andreani C, Raulet E, Pichard-Garcia L, Maurel P (2010) Use of human hepatocytes to investigate blood coagulation factor. Methods Mol Biol 640: 431–445. 20645066 10.1007/978-1-60761-688-7_23

[22] Dubuisson J, Hsu HH, Cheung RC, Greenberg HB, Russell DG, et al (1994) Formation and intracellular localization of hepatitis C virus envelope glycoprotein complexes expressed by recombinant vaccinia and Sindbis viruses. J Virol 68: 6147–6160. 8083956 10.1128/jvi.68.10.6147-6160.1994 PMC237034

[23] Flint M, Maidens C, Loomis-Price LD, Shotton C, Dubuisson J, et al (1999) Characterization of hepatitis C virus E2 glycoprotein interaction with a putative cellular receptor, CD81. J Virol 73: 6235–6244. 10400713 10.1128/jvi.73.8.6235-6244.1999 PMC112700

[24] Jones CT, Catanese MT, Law LM, Khetani SR, Syder AJ, et al (2010) Real-time imaging of hepatitis C virus infection using a fluorescent cell-based reporter system. Nat Biotechnol 28: 167–171. 20118917 10.1038/nbt.1604 PMC2828266

[25] Delgrange D, Pillez A, Castelain S, Cocquerel L, Rouillé Y, et al (2007) Robust production of infectious viral particles in Huh-7 cells by introducing mutations in HCV structural proteins. J Gen Virol 88: 2495–2503. 17698659 10.1099/vir.0.82872-0

[26] Goueslain L, Alsaleh K, Horellou P, Roingeard P, Descamps V, et al (2010) Identification of GBF1 as a cellular factor required for hepatitis C virus RNA replication. J Virol 84: 773–787. 19906930 10.1128/JVI.01190-09 PMC2798365

[27] Helle F, Vieyres G, Elkrief L, Popescu CI, Wychowski C, et al (2010) Role of N-linked glycans in the functions of hepatitis C virus envelope proteins incorporated into infectious virions. J Virol 84: 11905–11915. 20844034 10.1128/JVI.01548-10 PMC2977866

[28] Castelain S, Descamps V, Thibault V, Francois C, Bonte D, et al (2004) TaqMan amplification system with an internal positive control for HCV RNA quantitation. J Clin Virol 31: 227–234. 15465417 10.1016/j.jcv.2004.03.009

[29] Descamps V, Op de Beeck A, Plassart C, Brochot E, Francois C, et al (2012) Strong Correlation between Liver and Serum Levels of Hepatitis C Virus Core Antigen and RNA in Chronically Infected Patients. J Clin Microbiol 50: 465–468. 22162563 10.1128/JCM.06503-11 PMC3264181

[30] Vausselin T, Calland N, Belouzard S, Descamps V, Douam F, et al.. (2013) The antimalarial ferroquine is an inhibitor of hepatitis C virus. Hepatology: in press. 10.1002/hep.26273 PMC7165689 23348596

[31] Pokrovskii MV, Bush CO, Beran RK, Robinson MF, Cheng G, et al (2011) Novel mutations in a tissue culture-adapted hepatitis C virus strain improve infectious-virus stability and markedly enhance infection kinetics. J Virol 85: 3978–3985. 21289124 10.1128/JVI.01760-10 PMC3126117

[32] Zhong J, Gastaminza P, Chung J, Stamataki Z, Isogawa M, et al (2006) Persistent hepatitis C virus infection in vitro: coevolution of virus and host. J Virol 80: 11082–11093. 16956932 10.1128/JVI.01307-06 PMC1642175

[33] Zeisel MB, Fofana I, Fafi-Kremer S, Baumert TF (2011) Hepatitis C virus entry into hepatocytes: molecular mechanisms and targets for antiviral therapies. J Hepatol 54: 566–576. 21146244 10.1016/j.jhep.2010.10.014

[34] Timpe JM, Stamataki Z, Jennings A, Hu K, Farquhar MJ, et al (2008) Hepatitis C virus cell-cell transmission in hepatoma cells in the presence of neutralizing antibodies. Hepatology 47: 17–24. 17941058 10.1002/hep.21959

[35] Gottwein JM, Scheel TK, Hoegh AM, Lademann JB, Eugen-Olsen J, et al (2007) Robust hepatitis C genotype 3a cell culture releasing adapted intergenotypic 3a/2a (S52/JFH1) viruses. Gastroenterology 133: 1614–1626. 17983807 10.1053/j.gastro.2007.08.005

[36] Kang JI, Kim JP, Wakita T, Ahn BY (2009) Cell culture-adaptive mutations in the NS5B gene of hepatitis C virus with delayed replication and reduced cytotoxicity. Virus Res 144: 107–116. 19374927 10.1016/j.virusres.2009.04.002

[37] Takeda M, Ikeda M, Ariumi Y, Wakita T, Kato N (2012) Development of hepatitis C virus production reporter-assay systems using two different hepatoma cell lines. J Gen Virol 93: 1422–1431. 22456614 10.1099/vir.0.040725-0

[38] Fournier C, Helle F, Descamps V, Morel V, Francois C, et al.. (2013) Natural selection of adaptive mutations in non-structural genes increases trans-encapsidation of hepatitis C virus replicons lacking envelope protein genes. J Gen Virol: in press. 10.1099/vir.0.049676-0 23288424

[39] Pacini L, Graziani R, Bartholomew L, De Francesco R, Paonessa G (2009) Naturally occurring hepatitis C virus subgenomic deletion mutants replicate efficiently in Huh-7 cells and are trans-packaged in vitro to generate infectious defective particles. J Virol 83: 9079–9093. 19587042 10.1128/JVI.00308-09 PMC2738267

[40] Marukian S, Andrus L, Sheahan TP, Jones CT, Charles ED, et al (2011) Hepatitis C virus induces interferon-lambda and interferon-stimulated genes in primary liver cultures. Hepatology 54: 1913–1923. 21800339 10.1002/hep.24580 PMC3219820

[41] Park H, Serti E, Eke O, Muchmore B, Prokunina-Olsson L, et al (2012) IL-29 is the dominant type III interferon produced by hepatocytes during acute hepatitis C virus infection. Hepatology 56: 2060–2070. 22706965 10.1002/hep.25897 PMC3581145

[42] Thomas E, Gonzalez VD, Li Q, Modi AA, Chen W, et al (2012) HCV infection induces a unique hepatic innate immune response associated with robust production of type III interferons. Gastroenterology 142: 978–988. 22248663 10.1053/j.gastro.2011.12.055 PMC3435150

[43] Witte K, Witte E, Sabat R, Wolk K (2010) IL-28A, IL-28B, and IL-29: promising cytokines with type I interferon-like properties. Cytokine Growth Factor Rev 21: 237–251. 20655797 10.1016/j.cytogfr.2010.04.002

[44] Kambara H, Fukuhara T, Shiokawa M, Ono C, Ohara Y, et al (2012) Establishment of a Novel Permissive Cell Line for the Propagation of Hepatitis C Virus by Expression of MicroRNA miR122. J Virol 86: 1382–1393. 22114337 10.1128/JVI.06242-11 PMC3264374

[45] Narbus CM, Israelow B, Sourisseau M, Michta ML, Hopcraft SE, et al (2011) HepG2 cells expressing microRNA miR-122 support the entire hepatitis C virus life cycle. J Virol 85: 12087–12092. 21917968 10.1128/JVI.05843-11 PMC3209320

[46] Sainz B Jr, Barretto N, Yu X, Corcoran P, Uprichard SL (2012) Permissiveness of human hepatoma cell lines for HCV infection. Virol J 9: 30. 22273112 10.1186/1743-422X-9-30 PMC3317838

[47] Thibault PA, Huys A, Dhillon P, Wilson JA (2013) MicroRNA-122-dependent and -independent replication of Hepatitis C Virus in Hep3B human hepatoma cells. Virology 436: 179–190. 23245472 10.1016/j.virol.2012.11.007

[48] Han Q, Xu C, Wu C, Zhu W, Yang R, et al (2009) Compensatory mutations in NS3 and NS5A proteins enhance the virus production capability of hepatitis C reporter virus. Virus Res 145: 63–73. 19540283 10.1016/j.virusres.2009.06.005

[49] Kaul A, Woerz I, Meuleman P, Leroux-Roels G, Bartenschlager R (2007) Cell culture adaptation of hepatitis C virus and in vivo viability of an adapted variant. J Virol 81: 13168–13179. 17881454 10.1128/JVI.01362-07 PMC2169131

[50] Russell RS, Meunier JC, Takikawa S, Faulk K, Engle RE, et al (2008) Advantages of a single-cycle production assay to study cell culture-adaptive mutations of hepatitis C virus. Proc Natl Acad Sci USA 105: 4370–4375. 18334634 10.1073/pnas.0800422105 PMC2393785

[51] Scheel TK, Gottwein JM, Jensen TB, Prentoe JC, Hoegh AM, et al (2008) Development of JFH1-based cell culture systems for hepatitis C virus genotype 4a and evidence for cross-genotype neutralization. Proc Natl Acad Sci U S A 105: 997–1002. 18195353 10.1073/pnas.0711044105 PMC2242719

[52] Mee CJ, Grove J, Harris HJ, Hu K, Balfe P, et al (2008) Effect of cell polarization on hepatitis C virus entry. J Virol 82: 461–470. 17959672 10.1128/JVI.01894-07 PMC2224355

[53] Sainz B Jr, Barretto N, Uprichard SL (2009) Hepatitis C virus infection in phenotypically distinct Huh7 cell lines. PLoS One 4: e6561. 19668344 10.1371/journal.pone.0006561 PMC2720605

[54] Coulouarn C, Factor VM, Andersen JB, Durkin ME, Thorgeirsson SS (2009) Loss of miR-122 expression in liver cancer correlates with suppression of the hepatic phenotype and gain of metastatic properties. Oncogene 28: 3526–3536. 19617899 10.1038/onc.2009.211 PMC3492882

[55] Icard V, Diaz O, Scholtes C, Perrin-Cocon L, Ramiere C, et al (2009) Secretion of hepatitis C virus envelope glycoproteins depends on assembly of apolipoprotein B positive lipoproteins. PLoS One 4: e4233. 19156195 10.1371/journal.pone.0004233 PMC2617766

[56] Jammart B, Michelet M, Pecheur EI, Parent R, Bartosch B, et al.. (2013) VLDL-producing and HCV-replicating HepG2 cells secrete no more LVP than VLDL-deficient Huh7.5 cells. J Virol: in press. 10.1128/JVI.01405-12 PMC3624324 23427158

[57] Forte TM, McCall MR, Knowles BB, Shore VG (1989) Isolation and characterization of lipoproteins produced by human hepatoma-derived cell lines other than HepG2. J Lipid Res 30: 817–829. 2551986

