# Correction: Permissivity of Primary Human Hepatocytes and Different Hepatoma Cell Lines to Cell Culture Adapted Hepatitis C Virus

**DOI:** 10.1371/journal.pone.0223022

**Published:** 2019-09-19

**Authors:** Francois Helle, Etienne Brochot, Carole Fournier, Véronique Descamps, Laure Izquierdo, Thomas W. Hoffmann, Virginie Morel, Yves-Edouard Herpe, Abderrahmane Bengrine, Sandrine Belouzard, Czeslaw Wychowski, Jean Dubuisson, Catherine Francois, Jean-Marc Regimbeau, Sandrine Castelain, Gilles Duverlie

The cell line HepG2-CD81 appears incorrectly throughout the article [1]. The correct cell line is HuH-7. This correction is in line with the correction for Belouzard et al., “Entry and release of hepatitis C virus in polarized human hepatocytes,” [2] whose authors provided the cell line for analysis. Results concerning the HepG2-CD81 permissivity to HCV (Figure 6 in [1]) and the HCV virions production by HepG2-CD81 cells (Figure 7 in [1]) must be interpreted as permissivity of a HuH-7 clone to HCV and HCV virions production by a HuH-7 clone, respectively.

There are errors in Fig 6, “Infection of hepatoma cell lines with cell culture adapted HCV”, and Fig 7, “Profiles of density of HCV produced in different hepatoma cell lines”, which reference the HepG2-CD81 cell line. In addition, there are errors in the captions for Figs 6 and 7, which reference the HepG2-CD81 cell line. Please see the corrected Figs 6 and 7, and their corrected captions, here.

**Fig 6:**
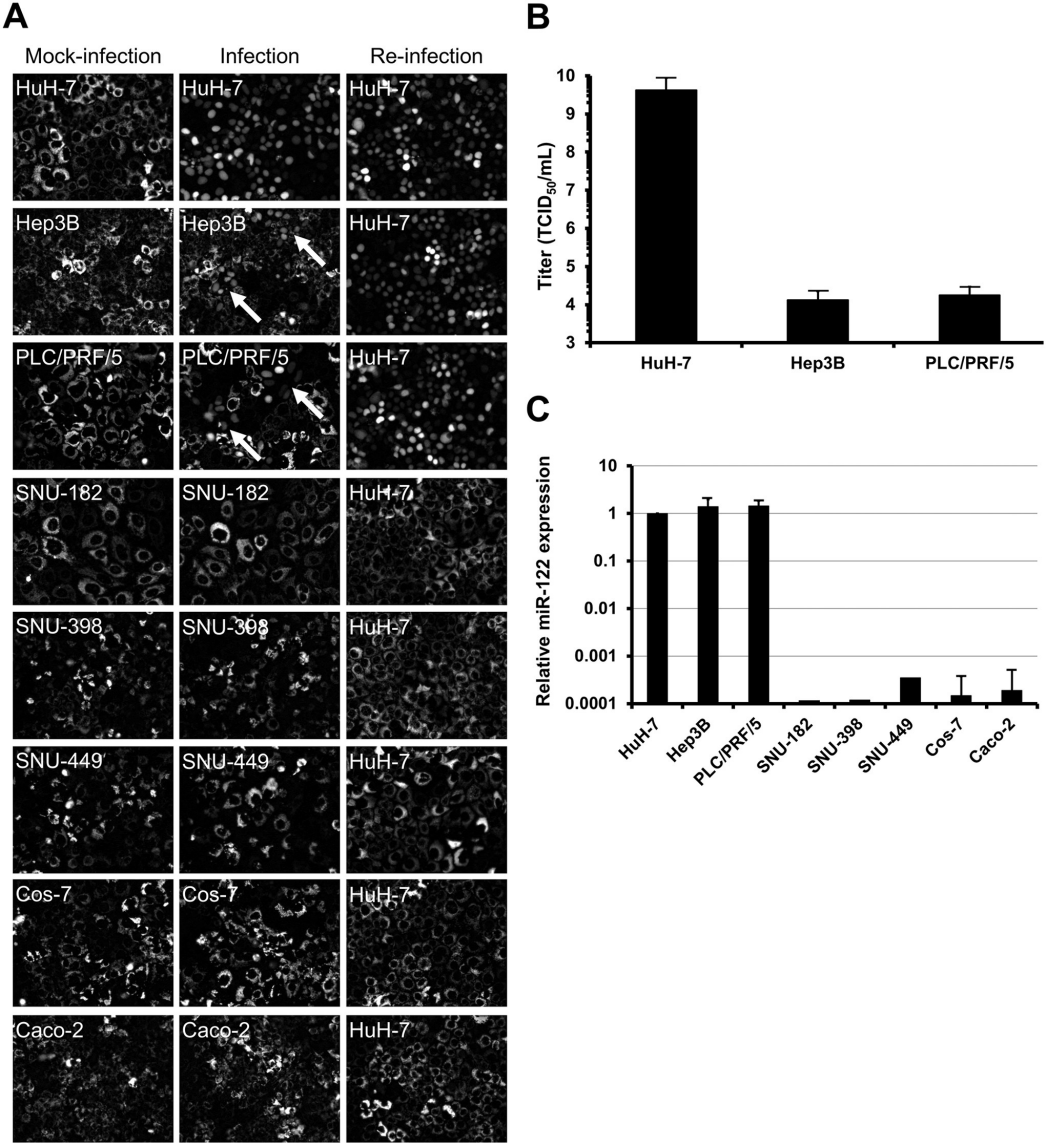
Infection of hepatoma cell lines with cell culture adapted HCV. (**A**) HuH-7, Hep3B, PLC/PRF/5, SNU-182, SNU-398, SNU-449, Cos-7 and Caco-2 cells transduced with lentivirus expressing RFP-NLS-IPS were mock-infected (left) or inoculated with i24 (middle) (MOI = 10000 HuH-7 infectious units per cell). Infected cells, identified by translocation of the cleavage product RFP-NLS to the nucleus, were visualized 48 h post-infection. The supernatants of inoculated cells were recovered 72 h post-infection, centrifuged and used to inoculate naive HuH- 7-RFP-NLS-IPS to check the production of progeny virus (right). Images are representative of three independent experiments. (**B**) The permissivity of HuH-7, Hep3B and PLC/PRF/5 cells to the cell culture adapted virus was determined by TCID_50_ assay. The results are expressed as TCID_50_/mL ± S.D. calculated on 8 wells. (**C**) miR-122 expression was determined by RT-qPCR in HuH-7, Hep3B, PLC/PRF/5, SNU-182, SNU-389, SNU-449, Cos-7 and Caco-2 cells. The results, which are representative of four independent experiments, are expressed as relative miR-122 expression using the ΔΔCt method with RNU6B as endogenous control and HuH-7 cells as calibrator.

**Fig 7:**
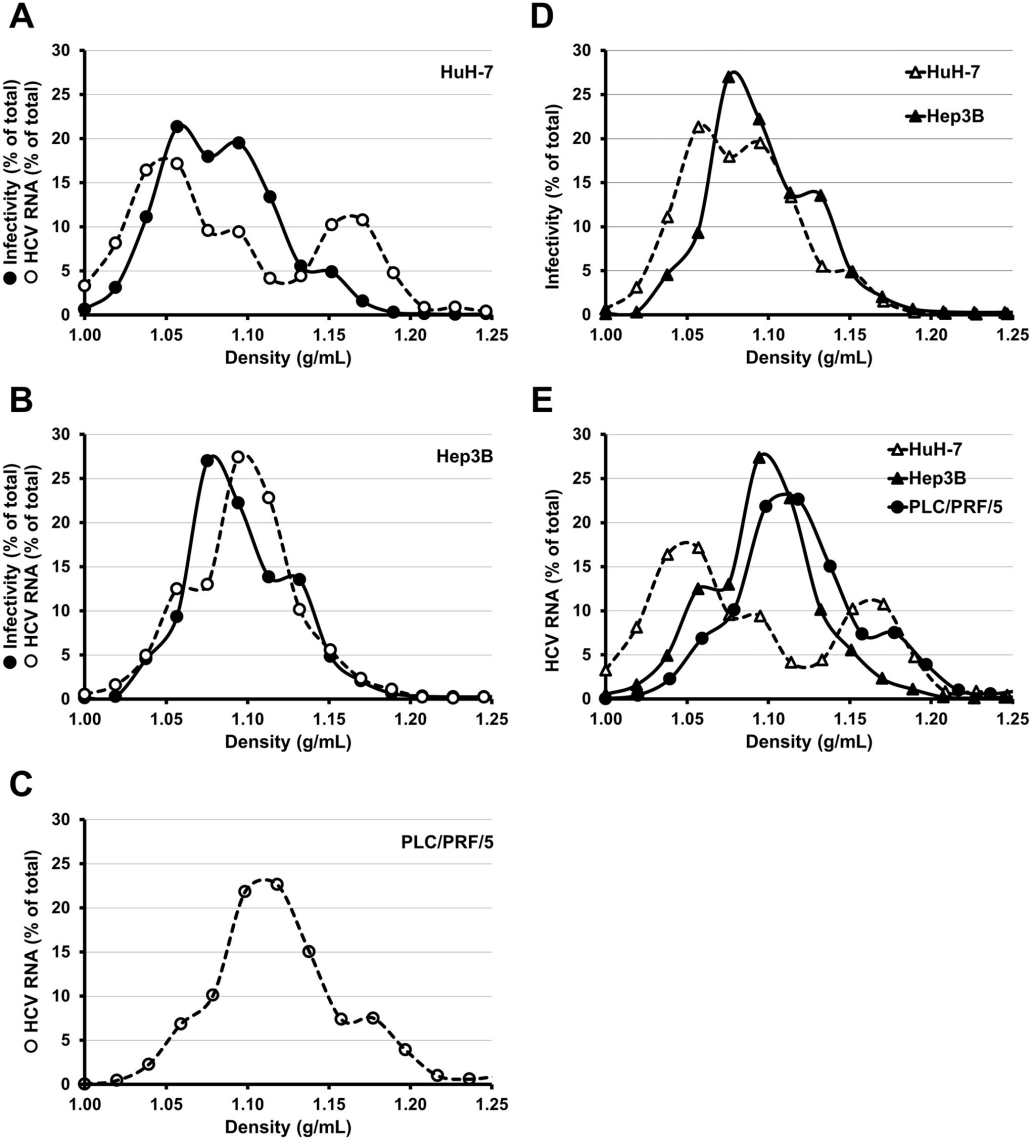
Profiles of density of HCV produced in different hepatoma cell lines. HuH-7 (**A**), Hep3B (**B**) and PLC/PRF/5 (**C**) were electroporated with in vitro transcribed RNA of the JFH1-CS-A4-RLuc genome containing mutations R1373Q/C2441S. The supernatants of each electroporated cell lines were recovered six days post-electroporation and overlaid on 10 to 50% (weight/volume) iodixanol gradients. After a 24 h ultracentrifugation, sixteen fractions were collected and analyzed for HCV RNA quantity and infectivity on naive HuH-7 cells (assessed by measuring Renilla Luciferase activities). The results are expressed as percentages of total infectivity or HCV RNA and are reported as means of two independent experiments.
